# An Optimal Spatio-Temporal Hybrid Model Based on Wavelet Transform for Early Fault Detection

**DOI:** 10.3390/s24144736

**Published:** 2024-07-21

**Authors:** Jingyang Xing, Fangfang Li, Xiaoyu Ma, Qiuyue Qin

**Affiliations:** 1 School of Chang Chien, Nantong University, Nantong 226019, China; xiaoxing@stmail.ntu.edu.cn (J.X.); xiaolee@stmail.ntu.edu.cn (F.L.); 2 School of Electrical Engineering and Automation, Nantong University, Nantong 226019, China; xiaoyuma@stmail.ntu.edu.cn

**Keywords:** wavelet transform, principal component analysis, kernel density estimation, spatio-temporal hybrid model, early fault detection

## Abstract

An optimal spatio-temporal hybrid model (STHM) based on wavelet transform (WT) is proposed to improve the sensitivity and accuracy of detecting slowly evolving faults that occur in the early stage and easily submerge with noise in complex industrial production systems. Specifically, a WT is performed to denoise the original data, thus reducing the influence of background noise. Then, a principal component analysis (PCA) and the sliding window algorithm are used to acquire the nearest neighbors in both spatial and time dimensions. Subsequently, the cumulative sum (CUSUM) and the mahalanobis distance (MD) are used to reconstruct the hybrid statistic with spatial and temporal sequences. It helps to enhance the correlation between high-frequency temporal dynamics and space and improves fault detection precision. Moreover, the kernel density estimation (KDE) method is used to estimate the upper threshold of the hybrid statistic so as to optimize the fault detection process. Finally, simulations are conducted by applying the WT-based optimal STHM in the early fault detection of the Tennessee Eastman (TE) process, with the aim of proving that the fault detection method proposed has a high fault detection rate (FDR) and a low false alarm rate (FAR), and it can improve both production safety and product quality.

## 1. Introduction

Effective early fault detection in the industrial production process is extremely important for improving the operation safety of production systems and achieving the maximum economic benefit. In modern industrial processes [1], all the links are correlated. In particular, in chemistry [2], petroleum [3], pharmacies [4], and other crucial sectors, the failure of any link will lead to the failure of partial functions in the production process or even the entire process. Currently available fault detection systems cannot effectively process the dynamic data produced by the changes in high-frequency operations of the system and the complex spatial correlation between production links. In addition, these systems often fail to detect and respond to early tiny changes or abnormalities because they lack an advanced data processing method that can comprehensively and synchronously analyze complex dynamic data. Therefore, effective fault detection of complex industrial processes can improve industrial production safety. Developing new technology to improve early fault detection precision and response speeds has become a research focus in this field.

Data-driven fault detection and process monitoring techniques, such as principal component analysis (PCA) [5], partial least squares (PLSs) [6], and independent component analysis (ICA) [7], have been used to monitor complex chemical reactions and process control systems in the chemical industrial process [8]. These methods extract the main or independent components from the key variables and use Hotelling’s T-square ($T^{2}$) [9] and the square prediction error (SPE, also called the Q statistic) [10] for production process monitoring and quality control. However, these statistics are usually based on the assumption that the data come from an independent and identically distributed unimodal production environment [11]. This assumption may not hold in a multimodal process [12]. For instance, when the production conditions change, a single monitoring model will not be able to reflect correctly the dynamic changes in all the operation states [13]. In addition, these methods often do not take nonlinear relationships and complex correlations between time sequences into account, which may lead to the delayed or missed detection of faults.

There are multiple variables involved in the fault detection of modern industrial systems, so the dynamic analysis method has been applied to the real-time monitoring of key parameters in the industrial production process [14]. Zhou et al. proposed a hierarchical PCA method based on differential features for dynamic fault detection and used it to identify abnormalities in the production process [15]. Pan et al. developed a generalized likelihood ratio (GLR)-based fault detection method for non-Gaussian dynamic processes [16]. Song et al. made a dynamic inner slow feature analysis and proposed a method based on feature selection and extraction for enhancing the sensitivity of fault detection in walking gear systems [17]. However, the performance of the above-mentioned methods may be affected by the changes in the actual process. In particular, when they are used for mean-shift detection, delays are likely to occur, and they often cannot detect the fault accurately in time [18].

To solve the problem of nonlinear features in the fault detection of modern industrial systems, Mohammed et al. designed an expanded Kalman fault detection method specifically for nonlinear random systems [19]. Ferdowsi improved this method and introduced fault detection and prediction techniques for actuators and sensors applicable to multidimensional nonlinear partial differential equation systems [20]. Moreover, Han et al. proposed a new fault detection method based on nonlinear factorization and fuzzy models, which could identify early faults that caused data distribution changes [21]. Yan and Zhang et al. analyzed the hierarchical structure of the nonlinear system and built an unmeasurable nonlinear system fault detection framework [22]. Furthermore, Gong et al. used multi-source information fusions in the fault detection of nonlinear systems [23]. This method can effectively detect early faults in chemical processes.

With the development of machine learning in the artificial intelligence field, it has been applied to fault detection and prediction in modern industrial systems [24]. Harichandran et al. used hybrid machine learning frameworks to recognize device activity and detect early faults in automated construction [25]. Wang et al. employed graph autoencoders and ensemble learning for the fault detection of bearings [26]. Shubita et al. proposed a method for fault detection in rotating machines based on sound signals using edge machine learning [27]. Kumar et al. summarized research advances in the machine learning algorithm-based fault detection of asynchronous motors [28]. The above-mentioned learning algorithms perform well in early fault detection, but they require a large training dataset, which is difficult to obtain in practical situations [29]. In addition, deep learning models have high computational complexity, requiring significant computational resources for training and inference, which makes it difficult to meet the demands of real-time detection [30,31]. The interpretability of deep learning models is also low, making the diagnostic results challenging for industrial engineers to understand and apply.

New statistic models have been developed for data analysis and to improve detection sensitivity and accuracy. Hou et al. introduced a spatial data processing technique into the spatial geometry-based fault detection of output feedback systems, aiming at solving the problems that traditional methods encounter when processing complex data [32]. Qian et al. developed a method of fault detection in wind turbines based on spatio-temporal features and neighborhood operation states [33]. Temporal sequence analysis and spatial analysis improved the ability to predict the fault development trend. However, some of these methods cannot acquire the nonlinear relationship between large-scale datasets and systems in complex industrial processes, and some cannot solve the complex correlations between temporal sequences. Moreover, their generalization ability and reliability in practical applications still require verification.

Therefore, a wavelet transform (WT)-based optimal spatio-temporal hybrid model (STHM) is proposed for the high-sensitivity and high-precision detection of early faults in complex industrial production systems [34]. A WT is made first to denoise the data and reduce the influence of background noise. PCA, cumulative sum (CUSUM), and mahalanobis distance (MD) methods are then used to reconstruct the STHM with temporal and spatial sequences [35,36,37]. Moreover, the kernel density estimation (KDE) method is used to optimize the detection process, thereby enhancing the correlation between high-frequency temporal dynamics and space and improving fault detection precision. Simulations are made on the Tenessee Eastman (TE) experimental platform to compare the fault detection performance of the proposed model with that of the PCA and spatio-temporal nearest neighbor (STN) methods [38,39]. This study aims to demonstrate that the proposed method has a high FDR and a low FAR, and it can improve production safety and product quality.

## 2. Preliminary Work

### 2.1. Wavelet Transform

Data collected from chemical processes are often complex and dynamic, with high-dimensional and complex temporal sequences. WT is effective in the analysis of signals at different scales since it has the characteristics of time-frequency localization. Therefore, WT is able to preserve more key information in dynamic processes while reducing the impact of noise. During the WT process, signal energy is mainly concentrated in some coefficients, and noise energy is distributed on the entire coefficient axis. The wavelet coefficient of signals is generally larger than that of noise. These coefficients describe the energy distribution of signals at different scales. A noisy model is represented as follows:

(1) $$Z\left(i\right)=X\left(i\right)+\varepsilon *e\left(i\right)\;i=1,2...,n$$

where $X\left(i\right)$ is the real signal required, $Z\left(i\right)$ is the original signal with noise, $e\left(i\right)$ is noise, and ε is the variable coefficient of noise.

To differentiate real signals from noise, a corresponding threshold is set, and wavelet coefficients smaller than this threshold are removed. In this study, a soft threshold function is used. All the wavelet coefficients smaller than the set threshold are considered as noise and removed, and those larger than the threshold are retained and taken as important components of signals. The soft threshold function is the following:

(2) $$w_{j,k}=\left\{\begin{array}{cc} \mathrm{sgn}(w_{j,k})(\left|w_{j,k}\right|-thr) & \left|w_{j,k}\right|\geq thr\\ 0 & \left|w_{j,k}\right|<thr \end{array}\right.$$

where $w_{j,k}$ is the kth wavelet coefficient on the jth layer of WT, and thr is the threshold. A proper threshold can improve the noise reduction effect and help preserve key features. The threshold is calculated by the following:

(3) $$thr=\sigma \sqrt{2\ln N}$$

where σ is the standard deviation of noise after decomposition on the jth layer. It is estimated by the following:

(4) $$\sigma =\frac{1}{0.6745}\times \frac{1}{N}\sum_{k=1}^{N}\left|\omega_{j,k}\right|$$

**Figure 1:**
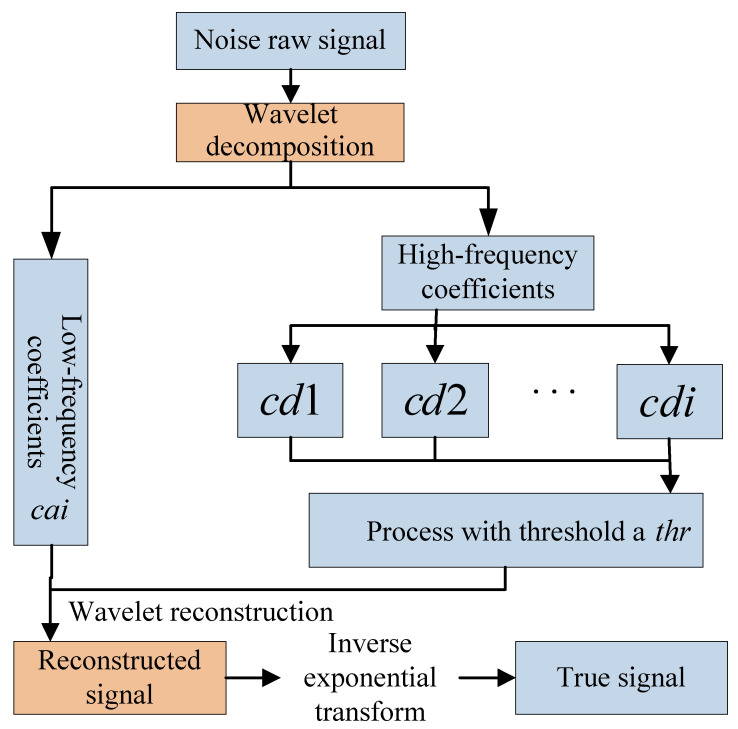
Wavelet denoising workflow diagram.

The real signal $X\left(i\right)$ obtained by WT provides more accurate and clear data support for subsequent fault detection and improves the reliability and accuracy of fault detection. The WT process is detailed in Figure 1.

As shown in Figure 1, there are several key steps for wavelet denoising:

1. The WT of the original signal is performed. In this process, the signal is decomposed to i layers, including an approximate component cai and detailed components $\left(cd1,cd2,...,cdi\right)$.
2. The detailed components are compared with the given threshold $\left(thr\right)$, and the signal components lower than this threshold are deemed noise and removed.
3. The processed detailed components and unprocessed approximate components are used to reconstruct the signal, which is then transformed to the time domain from the wavelet domain. The signal is, thus, finally restored.

### 2.2. Principal Component Analysis

In the fault detection of multivariate industrial processes, PCA is extensively applied to the dimensional reduction or feature extraction of data as it can effectively identify the most important components in data.

In this study, we assume that an industrial process has m observed variables, which are obtained by n times of data sampling. The data are preprocessed by WT and a new dataset $X\left(i\right)$ is acquired. The new dataset is divided into a training dataset $X^{tr}={\left[x_{1}^{tr},x_{2}^{tr},......,x_{n}^{tr}\right]}^{T}\in R^{n\times m}$ and a testing dataset $X^{te}={\left[x_{1}^{te},x_{2}^{te},......,x_{n}^{te}\right]}^{T}\in R^{n\times m}$. The training dataset $X^{tr}$ and testing dataset $X^{te}$ are standardized into n×m dimensional matrices $X_{nm}^{tr}$ and $X_{nm}^{te}$, respectively. The matrices are normalized to eliminate the adverse effect of primary variables in the dimensional reduction process. The dispersion of m columns of data is measured according to the correlation between two features calculated by the covariance formula. The m×m dimensional covariance matrix $C_{mm}$ constructed for the calculation of the correlation between two of each m features are indicated by the following:

(5) $$C_{mm}=\frac{1}{n-1}(X_{nm}-{\overset{⎯}{X}}_{nm}{)}^{T}(X_{nm}-{\overset{⎯}{X}}_{nm})$$

The eigenvalue $\lambda_{m}$ and eigenvector $p_{m}$ of $C_{mm}$ are solved. By arranging eigenvalues $\lambda_{m}$ according to the size, we can obtain corresponding eigenvectors $p_{m}$ and generate a matrix $P_{m}$. Supposing that m dimensional eigenvectors are reduced to k dimensional eigenvectors, the first k columns of the load matrix are selected to construct a new load matrix $P_{mk}$. Then, $X_{nm}$ is reconstructed, and a score matrix is obtained. That is, the original dataset is projected onto the space formed by k eigenvectors. The PCA model is finally obtained as follows:

(6) $$X=X_{nm}P_{mk}+E$$

where $X_{nm}P_{mk}$ is the principal subspace and E is the residual subspace.

The principal components are selected according to the cumulative percent variance (CPV). An expected CPV no smaller than 85% is given at first. The first k eigenvalues of $C_{mm}$ are selected, and the ratio of the sum of these eigenvalues to the sum of all eigenvalues reflects the CPV. The k value is determined when the CPV calculated is greater than the given value for the first time. The calculation formula for CPV is as follows:

(7) $$CPV(k)=\frac{\sum_{j=1}^{k}\lambda_{j}}{\sum_{i=1}^{m}\lambda_{i}}\times 100\%\geq 90\%$$

In this study, the PCA method is used to process the training dataset $X_{nm}^{tr}$. The load matrix $P_{mk}$ derived from Formulas (5)–(7) effectively extracts the most important variations in the data. These principal components are used to reconstruct the testing dataset $X_{nm}^{te}$, and finally, the principal subspace $N\left(x\right)\in R^{n\times k}$ is obtained. The PCA method optimizes data processing and can effectively identify and analyze key features in complex data.

### 2.3. Spatio-Temporal Nearest Neighbor Method for Fault Detection

A sliding window W is given to find the nearest neighbors $T\left(x\right)=\left\{x_{1},...,x_{f},...,x_{W}\right\}\in R^{W\times k}$ of the sample x in the principal subspace $N\left(x\right)$ for the time dimension. The k-nearest neighbors $Q\left(x\right)=\left\{x_{1},x_{2},...,x_{K}\right\}\in R^{K\times k}$ of the sample $x_{f}$ in the spatial dimension are searched based on distance. The mean and standard deviation of $Q\left(x\right)$ are calculated by the following:

(8) $$m\left(Q\left(x\right)\right)=\frac{1}{K}\sum_{f=1}^{K}x_{f}^{\prime }$$

(9) $$s\left(Q\left(x\right)\right)=\sqrt{\frac{1}{K-1}\sum_{f=1}^{K}{\left(x_{f}^{\prime }-m\left(Q\left(x\right)\right)\right)}^{2}}$$

The sample x is standardized:

(10) $$\overset{⎯}{x}=\frac{1}{W}\sum_{i=1}^{W}\frac{x-m\left(Q\left(x\right)\right)}{s\left(Q\left(x\right)\right)}$$

Euclidean distance is used to measure the distance between two points:

(11) $$d\left(x,y\right)=\sqrt{\sum_{i=1}^{n}{\left(x_{i}-y_{i}\right)}^{2}}$$

where x and y are two n dimensional points, and i is the dimension of data. The distance between each sample in the dataset and its k-nearest neighbors is calculated and used to estimate the local density of the sample. The distance is calculated by the following:

(12) $$D_{k}\left(x\right)=\frac{1}{k}\sum_{i=1}^{k}d\left(x,x_{\left(i\right)}\right)$$

where $x_{\left(i\right)}$ is the ith nearest neighbor of the sample x. All $D_{k}\left(x\right)$ values are arranged in order, and a high percentile p (of 0.95) is taken as the control limit. Data points with the highest estimated local density are considered a potential abnormality. The threshold is calculated by the following:

(13) $$STN=D_{k}^{\left(p\right)}$$

where $D_{k}^{\left(p\right)}$ is the pth percentile of all $D_{k}\left(x\right)$ values. During the monitoring process, the $D_{k}\left(x_{new}\right)$ value of a new observed point is calculated and compared with the control limit STN to determine if a malfunction occurs.

## 3. Spatio-Temporal Hybrid Model for Early Fault Detection

### 3.1. Construction of the Spatio-Temporal Hybrid Model

A sliding window W is given to find the nearest neighbors $T\left(x\right)=\left\{x_{1},x_{2},...,x_{W}\right\}\in R^{W\times k}$ of the sample x in the principal subspace $N\left(x\right)$ in the time dimension. The k-nearest neighbors $S\left(x\right)=\left\{x_{1},x_{2},...,x_{K}\right\}\in R^{K\times k}$ in the spatial dimension are searched based on distance.

In data analysis, especially in the monitoring of industrial processes or system state changes, CUSUM is a commonly used algorithm for the detection of minor changes. CUSUM is a sequential detection technology that accumulates incremental changes in data by recursion. The cumulative sum obtained can be used to update and detect statistical deviations in the data flow in real-time and effectively capture small changes or abnormalities in a process. The CUSUM formula for each variable is defined as follows:

(14) $$CUSUM_{ij}=max\left(0,CUSUM_{i-1,j}+(x_{ij}-\mu_{j}-c)\right)$$

where $CUSUM_{ij}$ and $x_{ij}$ are the CUSUM and observed values of the jth variable in the ith sample, respectively; $\mu_{j}$ is the expected offset value of the jth variable; and c is the decision interval.

The decision interval has great influence on the sensitivity and FAR of the CUSUM algorithm. To improve the performance of the algorithm, the standard deviation σ of the nearest neighbor sample set in the time dimension is estimated at first. A coefficient r is given, and the sensitivity of CUSUM is measured. The decision interval is calculated by the formula below to determine the change threshold of the statistic.

(15) c=r×σ

According to Formulas (14) and (15), for a given time window W, the statistic $S_{t}$ of the sample x in the time dimension is expressed as follows:

(16) $$S_{t}=\frac{1}{W}\sum_{i=1}^{W}\sum_{j=1}^{k}CUSUM_{ij}$$

The mean $\mu \left(x\right)=\frac{1}{K}\sum_{i=1}^{K}x_{i}$ and covariance matrix ∑ of the nearest neighbor set $S\left(x\right)$ of the sample x in the spatial dimension are obtained. 

First, data points are rotated, and dimensions are linearly independent. Then, we obtain the following:

(17) $$F=S\left(x\right)=U^{T}X$$

Since the dimensions are linearly independent after transformation and the eigenvalue of each dimension is its variance, we obtain the following:

(18) $$\left(F-\mu \right){\left(F-\mu \right)}^{T}=U^{T}\sum U$$

Through the rotation and scaling of Euclidean distance, MD is acquired as follows:

(19) $$D(x)=\sqrt{{\left(x-\mu \right)}^{T}\sum^{-1}\left(x-\mu \right)}$$

The statistic $S_{s}$ of the sample x in the spatial dimension is derived from Formulas (17) to (19):

(20) $$S_{s}=D_{\left(x\right)}^{2}={\left(x-\mu \right)}^{T}\sum^{-1}\left(x-\mu \right)$$

### 3.2. Calculation of the Hybrid Statistic Using the Absolute Deviation

The absolute deviation can improve the sensitivity of the model to data changes in time and spatial dimensions, especially when statistics are related in time. The absolute deviation is defined as follows:

(21) $$M_{i}=\frac{1}{n}\sum_{i=1}^{n}\left|S_{t}\left(x_{i}\right)-S_{s}\left(x_{i}\right)\right|$$

where $M_{i}$ is the absolute deviation of the statistics of the ith sample in time and spatial dimensions. The basic weight of the time dimension is thus obtained as follows:

(22) $$\omega_{i}=\frac{S_{t}\left(x_{i}\right)}{M_{i}}$$

Through scaling, the ratio of statistics in different dimensions of the STHM enables the measurement of the maximum fault severity is obtained. The final hybrid statistic $S_{ts}$ is expressed as follows:

(23) $$S_{ts}\left(x_{i}\right)=\omega_{i}\cdot S_{t}\left(x_{i}\right)+\left(1-\omega_{i}\right)\cdot S_{s}\left(x_{i}\right)$$

The KDE method can flexibly and accurately estimate the distribution density of complex data and improve fault detection accuracy and reliability. A significance level of α=0.05 is given, and the upper limit of $S_{ts}$ is measured using the KDE method:

(24) $$\int \begin{array}{l} \eta_{S_{ts}}\left(\alpha \right)\\ -\infty \end{array}p\left(S_{ts}\right)d\left(S_{ts}\right)=\alpha$$

where $p\left(z\right)$ is the probability density function of a random variable z. The following fault detection logic is used in practice as follows:

(25) $$\left\{\begin{array}{cc} S_{ts}\left(x_{i}\right)\leq \eta_{S_{ts}}\left(\alpha \right),\forall i\in \left(1,2,...,n\right) & normal\\ S_{ts}\left(x_{i}\right)>\eta_{S_{ts}}\left(\alpha \right),\forall i\in \left(1,2,...,n\right) & fault \end{array}\right.$$

### 3.3. Steps of Fault Detection by the Spatio-Temporal Hybrid Model

**Figure 2:**
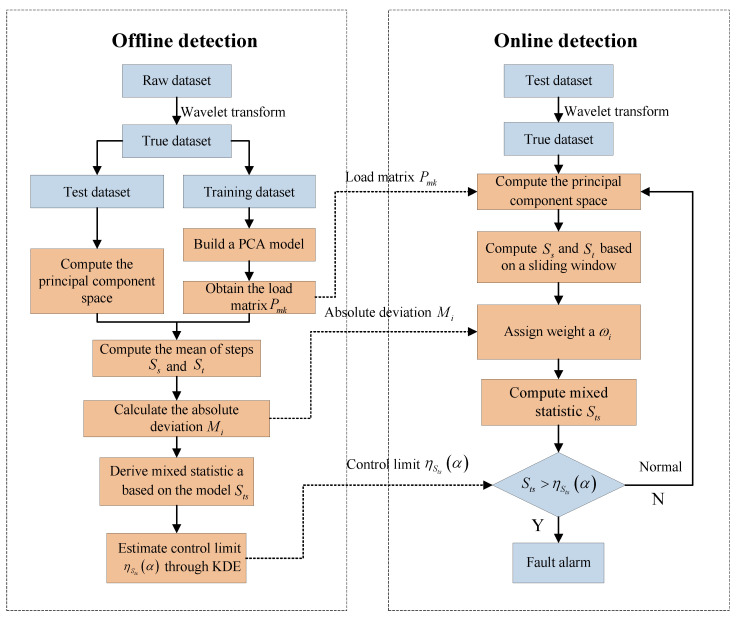
Flowchart of early fault detection based on the spatio-temporal hybrid model.

To solve the problem that the nearest neighbor set of the sample in the time dimension is incomplete, the window is extended forward to ensure that the sample set is complete. The fault detection flowchart based on the Spatiotemporal Hybrid Model (STHM) is shown in Figure 2. The fault detection process of the STHM is detailed as follows.

Offline detection

1. Normal samples are collected to form a dataset $Z\left(i\right)$ for the model, and a WT is conducted to reduce the influence of background noise. A real dataset $X\left(i\right)$ is thus obtained.
2. The real dataset is divided into a training dataset $X^{tr}={\left[x_{1}^{tr},x_{2}^{tr},......,x_{n}^{tr}\right]}^{T}\in R^{n\times m}$ and a testing dataset $X^{te}={\left[x_{1}^{te},x_{2}^{te},......,x_{n}^{te}\right]}^{T}\in R^{n\times m}$, which are standardized into n×m dimensional matrices $X_{nm}^{tr}$ and $X_{nm}^{te}$.
3. The PCA method is used to build a mode with a normal training dataset, and a load matrix ${P_{m}}_{k}$ is obtained. Through further calculation, the principal subspace $N\left(x\right)\in R^{n\times k}$ of the testing dataset is acquired.
4. A sliding window W is given to find the nearest neighbor set $T\left(x\right)=\left\{x_{1},x_{2},...,x_{W}\right\}$ of the sample x in the time dimension. The k-nearest neighbors $S\left(x\right)=\left\{x_{1},x_{2},...,x_{K}\right\}$ in the spatial dimension are searched based on distance. The statistics $S_{t}$ and $S_{s}\;$of the sample x in time and spatial dimensions are calculated, respectively.
5. The moving window method is used to repeat the last step. Statistics $S_{t}$ in n time dimensions and statistics $S_{s}$ in n spatial dimensions are obtained.
6. The absolute deviation $M_{i}$ is calculated, and the hybrid statistic $S_{ts}$ is obtained by the STHM.
7. A confidence level is given, and the control limit is estimated using the KDE method.

Online detection

1. Testing samples are collected, and they constitute a sample set $Z{\left(i\right)}^{\prime }$. Then, a WT is performed to obtain a real dataset $X{\left(i\right)}^{\prime }$ for online detection.
2. The load matrix ${P_{m}}_{k}$ obtained in offline detection is reconstructed, and the principal subspace $N{\left(x\right)}^{\prime }$ of the real dataset is obtained.
3. The moving window method is used to calculate statistics ${S_{t}}^{\prime }$ in the time dimension and ${S_{s}}^{\prime }$ in the spatial dimension.
4. The weight $\omega_{i}$ is calculated based on the absolute deviation, and then assigned to statistics ${S_{t}}^{\prime }$ in the time dimension and ${S_{s}}^{\prime }$ in the spatial dimension. The hybrid statistic ${S_{ts}}^{\prime }$ is finally acquired.
5. The control limit $\eta_{S_{ts}}\left(\alpha \right)$ obtained in offline detection is compared with the hybrid statistic ${S_{ts}}^{\prime }$ to determine if a fault occurs in the testing sample.

## 4. Experiment and Result Analysis of the TE Process

### 4.1. Simulation

**Figure 3:**
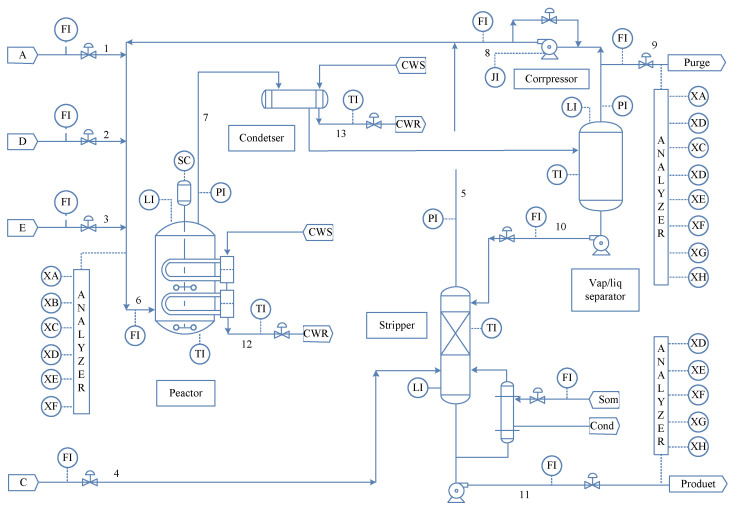
Process flow diagram of the TE process.

The TE process is a reliable simulation method mainly used in research on the control of chemical processes and also in multiple fields such as machinery equipment state estimation in production, the production state prediction model, and recognition of equipment failure sounds. Data produced by the platform are time-varying and nonlinear, with strong coupling strength, so it can adequately simulate typical features of real complex industrial process systems. The TE process mainly consists of five operating units, namely, a product condenser, a reactor, a product strip, a recycle compressor, and a vapor-liquid separator. The process flow diagram of the TE process is shown in Figure 3.

A total of 52 measurements, including 41 measured variables and 11 manipulated variables, were collected in the TE process. As shown in Figure 3, the measured variables included material flow, product pressure, reaction temperature, liquid level, etc. The manipulated variables are the open degrees of the valves. The degrees are set in the range of [0, 100], with 0 indicating close and 100 indicating fully open values. Table 1 introduces the main fault types of the TE process, including step change, random variations, the slow drift of variables, and sticky valves.

As shown in Table 1, in the TE process, a total of 21 faults were detected, which were classified into six types. Faults 1 to 7 are caused by step changes in process variables. Fault 4, which is the abnormal temperature of cooling water in the reactor, is a case in point. Faults 8 to 12 are attributed to random variations in process variables, such as Fault 12, which is the abnormal inlet temperature of cooling water in the condenser. Fault 13 refers to the abnormal constants of reaction kinetics, which are caused by slow changes in reaction kinetics. Faults 14 and 15 (which refer to the abnormal cooling water valve in the condenser) are triggered by sticky valves. Other faults are unknown types.

The dataset of the TE process simulates the real industrial production process. The 48 h-long simulation data were collected every 3 min, and fault data were introduced from the 8th hour. There was 1 normal state dataset and 21 fault datasets of different fault types. The data of each fault type were subdivided into a training set (containing 500 samples) and a testing set (containing 960 samples). To verify the performance of the WT-based STHM in fault detection, these were used to detect faults in the TE process, and their performance was compared to that of STN, T^2^, and SPE as part of the PCA method.

### 4.2. Signal Processing Model Demonstration

To analyze the effectiveness of the proposed model in signal processing, demonstrations were conducted using the training set of the TE dataset. The tenth feature column was selected for comparative analysis before and after wavelet transform.

**Figure 4:**
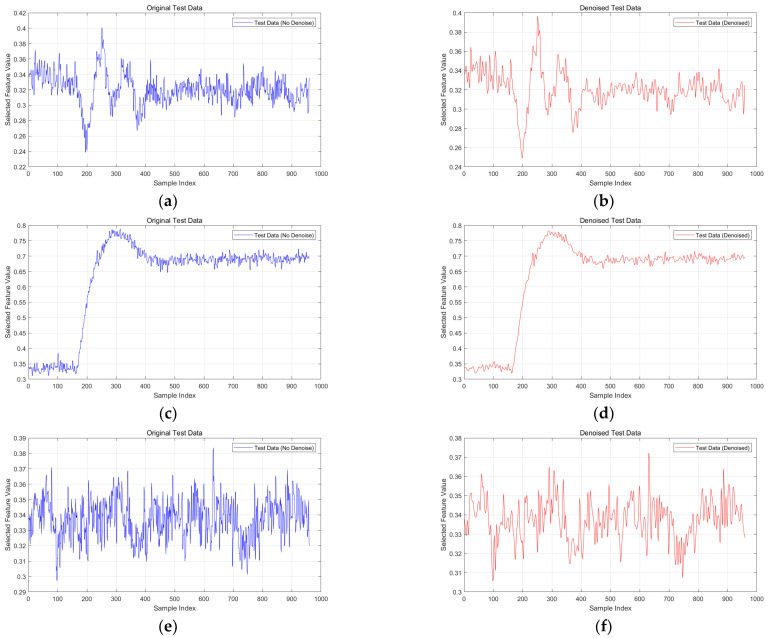

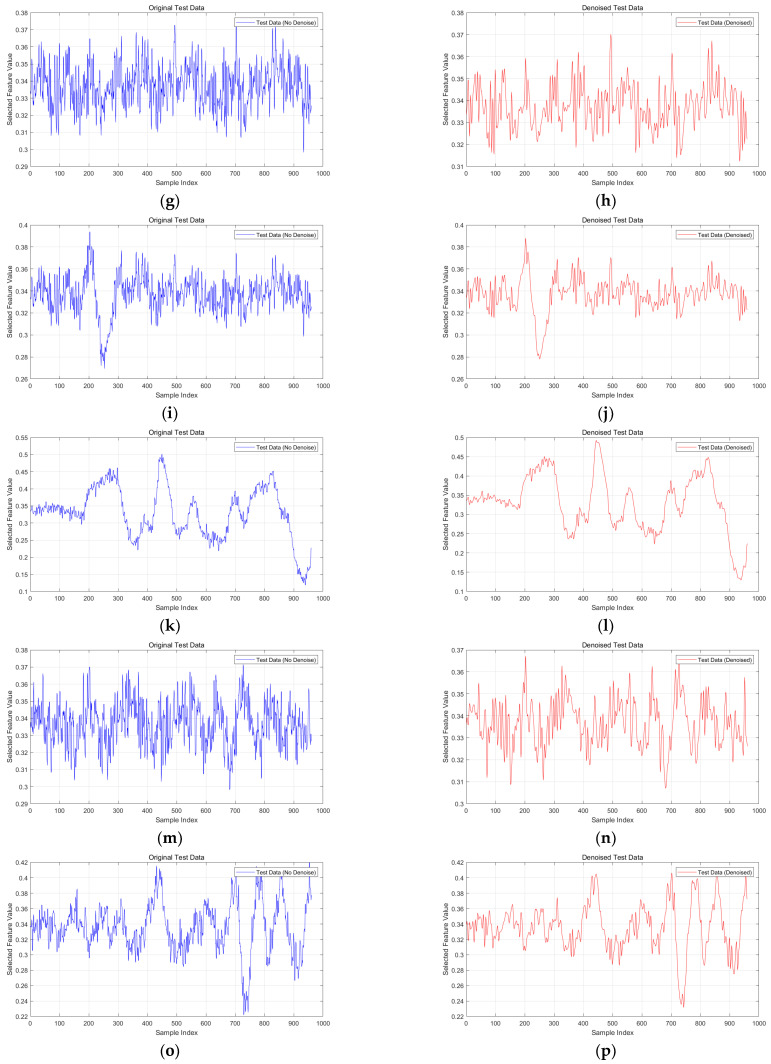

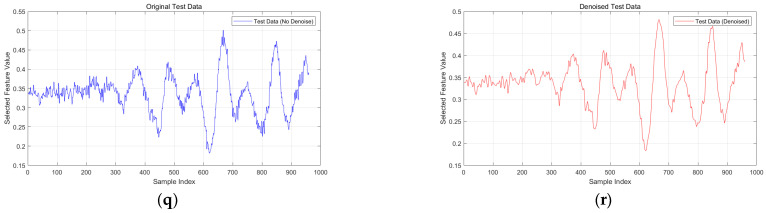
Detection results of statistical measures for Fault 4 using different detection methods. (**a**) Demonstration of raw data for Fault 1; (**b**) demonstration of denoised data for Fault 1; (**c**) demonstration of raw data for Fault 2; (**d**) demonstration of denoised data for Fault 2; (**e**) demonstration of raw data for Fault 3; (**f**) demonstration of denoised data for Fault 3; (**g**) demonstration of raw data for Fault 4; (**h**) demonstration of denoised data for Fault 4; (**i**) demonstration of raw data for Fault 5; (**j**) demonstration of denoised data for Fault 5; (**k**) demonstration of raw data for Fault 8; (**l**) demonstration of denoised data for Fault 8; (**m**) demonstration of raw data for Fault 10; (**n**) demonstration of denoised data for Fault 10; (**o**) demonstration of raw data for Fault 12; (**p**) demonstration of denoised data for Fault 12; (**q**) demonstration of raw data for Fault 13; and (**r**) demonstration of denoised data for Fault 13.

First, the raw data of the tenth feature column were analyzed. Next, the same feature column data were processed using wavelet transform. Wavelet transform, through multi-scale analysis, effectively separated the signal from the noise. Figure 4 shows the denoised signal before and after wavelet transform. It can be seen from the figure that wavelet transform reduces the noise, making the main features of the signal more prominent.

### 4.3. Simulation Result Analysis

**Figure 5:**
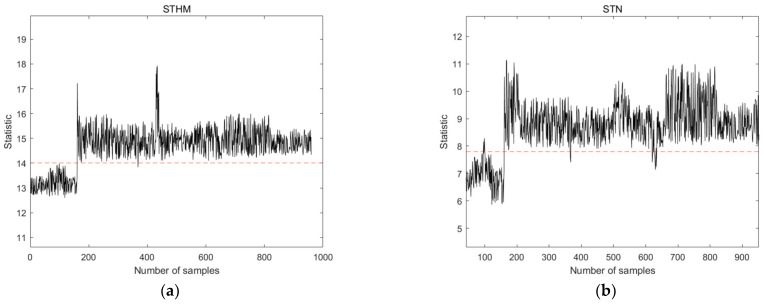

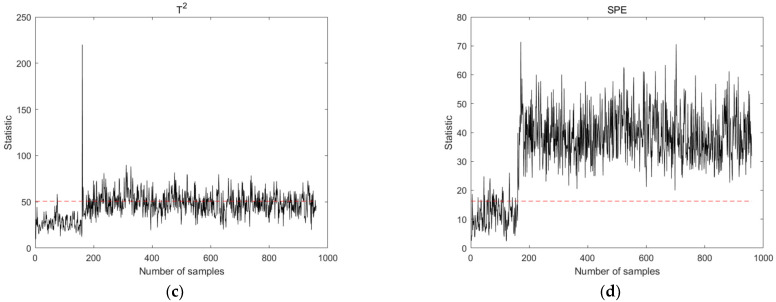
Detection results of statistical measures for Fault 4 using different detection methods. (**a**) Statistical measures of the STHM method; (**b**) statistical measures of the STN method; (**c**) $T^{2}$ statistics of the PCA method; and (**d**) SPE statistics of the PCA method.

**Figure 6:**
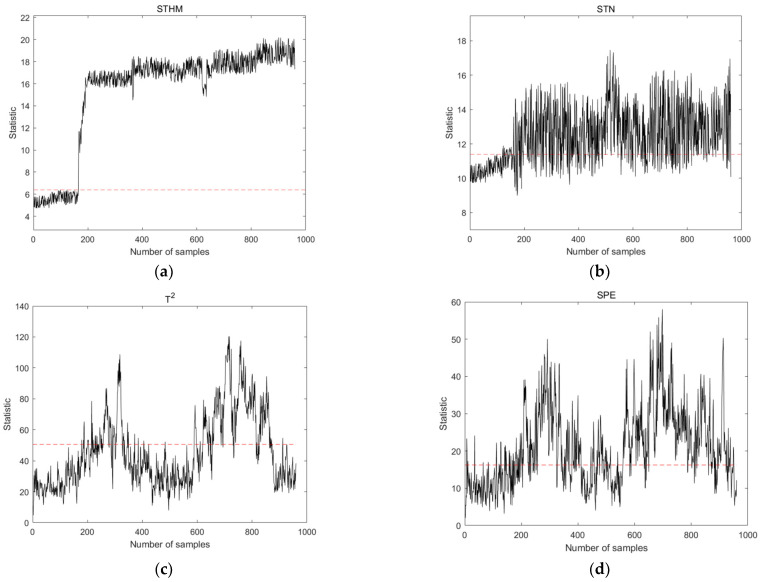
Detection results of statistical measures for Fault 10 using different detection methods. (**a**) Statistical measures of the STHM method; (**b**) statistical measures of the STN method; (**c**) $T^{2}$ statistics of the PCA method; and (**d**) SPE statistics of the PCA method.

**Figure 7:**
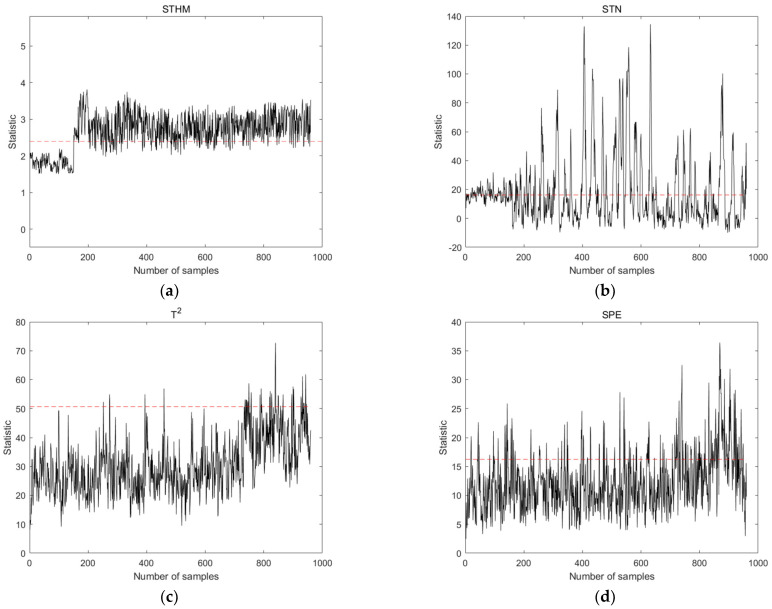
Detection results of statistical measures for Fault 15 using different detection methods. (**a**) Statistical measures of the STHM method; (**b**) statistical measures of the STN method; (**c**) $T^{2}$ statistics of the PCA method; and (**d**) SPE statistics of the PCA method.

Fault 4 (caused by step change), Fault 10 (caused by random variations), and Fault 15 (caused by sticky valves) were selected from the TE process for the comparison of the performance of STHM compared to that of the STN and PCA methods. These types of faults exhibited different characteristics in the early stages, which could fully demonstrate the comprehensive capability of detecting early faults. By analyzing the characteristics of the training dataset, the time window width was set to 8, and the length of the spatial nearest neighbor set was set to 10. The results are shown in Figure 5, Figure 6 and Figure 7. The dashed line in the figure represents the threshold line.

Figure 5 shows the results of Fault 4 detection using the four different methods. STHM responds fast and shows high sensitivity to the fault, especially in the early stage of the fault, due to its comprehensive consideration of the statistical information of time and space. Compared with STN, STHM shows better detection performance before and at the time of fault occurrence. Compared with the $T^{2}$ of the PCA method, STHM performs better when the fault is happening. STHM is superior to the SPE of the PCA method during detection performance and before fault occurrence.

Figure 6 shows the results of Fault 10 detection using the four different methods. Compared with other methods, STHM still exhibits excellent performance and can capture accurately and quickly respond to data fluctuations caused by random variations. STHM not only has a lower FAR than STN before fault occurrence but is also more accurate than STN in fault detection after fault occurrence. Compared with the $T^{2}$ and SPE of the PCA method, STHM can identify the fault earlier, with a lower FAR.

Figure 7 summarizes the results of Fault 15 detection using the four different methods. STHM has advantages over the other three methods in the accurate identification of early minor faults. STHM has a lower FAR and faster response speed than STN. STHM is able to detect the signs of fault occurrence earlier than the two statistic measures of the PCA method. In general, STHM can greatly enhance responses to early faults and reduce the FAR. It provides a more effective and practical technical means of detecting early faults. 

## 5. Discussion

Products FAR and FDR are often used as indicators of fault detection in industrial processes. In the experiment, the number of normal samples is labeled as TN, the number of fault samples FN, the number of false alarms $f_{n}$, and the number of samples for fault detection $t_{n}$. Product FAR, also called the false detection rate, refers to the probability that the statistic exceeds the threshold before fault occurrence. A low FAR indicates better detection performance. The FAR is expressed as follows:

(26) $$FAR=\frac{f_{n}}{TN}$$

FDR refers to the probability of false alarm rates when the fault occurs. In the TE process, FDR is the probability that the statistic exceeds the threshold at the time of fault occurrence. It is expressed as follows:

(27) $$FDR=\frac{t_{n}}{FN}$$

**Figure 8:**
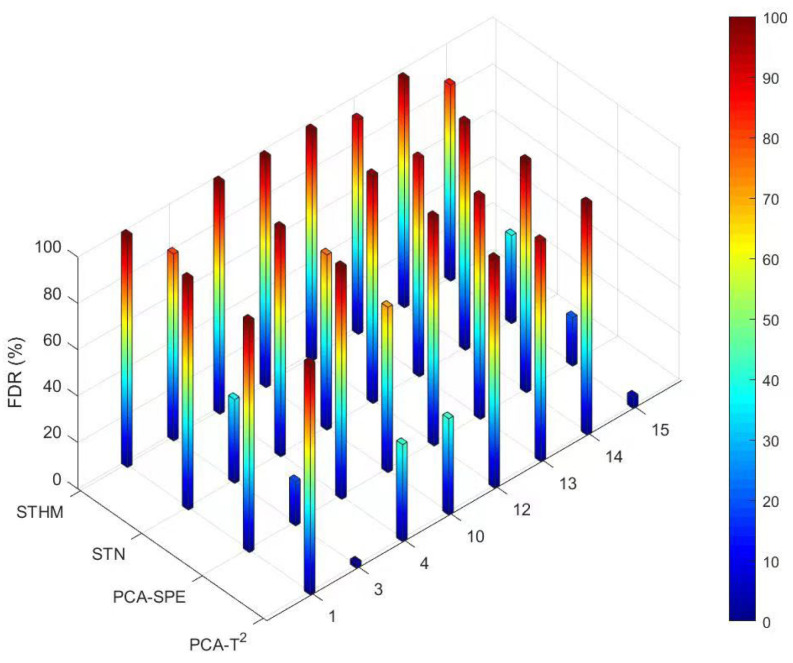
Comparison chart of FDR effects for different detection methods.

Eight different types of faults in the TE process were detected using the STHM, STN, and PCA methods. Their product FARs and FDRs were calculated, and the results are shown in Table 2. Figure 8 compares the FDRs of different methods. In the table, the bold numbers indicate that the product FARs and FDRs of STHM are better than those of the STN and PCA methods.

As shown in Table 2, STHM is superior to other methods in the detection of most types of faults. It has an excellent FDR and product FAR, indicating that it is highly sensitive to and can accurately identify early faults. Figure 8 compares the FDRs of different methods in the detection of different types of faults.

The FDRs of STHM for Faults 1 and 4 (both are caused by step change) are 99.8% and 99.9%, respectively, indicating the outstanding detection performance of STHM. The product FAR of STHM is 0%, demonstrating its higher reliability than the PCA and SPE methods. This is because STHM optimizes data denoising and feature extraction by integrating the WT with spatio-temporal data fusion. Therefore, STHM is more sensitive to and can accurately capture early step changes.

STHM also performs much better than other methods in the detection of Fault 10 (caused by random variations). It has an FDR of 99.4%, which is much higher than the 41.6% of PCA-$T^{2}$, 71.3% of PCA-SPE, and 75.6% of STN. This finding indicates that STHM can capture more accurately and respond more quickly to data fluctuations caused by random variations.

Moreover, STHM has a higher FDR in the detection of Faults 13 and 15 (caused by minor changes) in the principal component space compared with PCA and STN methods. It demonstrates that STHM has advantages in the detection of minor faults. Through the comprehensive analysis of time and space data, STHM shows improved sensitivity to tiny changes and can accurately detect the signs of faults in the early stage. Therefore, STHM enhances the early warning performance of the system. The product FAR of STHM is slightly higher than that of PCA-$T^{2}$. The reason for this may be that the calculations of CUSUM in the time dimension and its correlation in the spatial dimension increase the sensitivity of STHM to faults, leading to it responding to normal fluctuations or non-fault variations. The simulation results prove that STHM performs excellently in the detection of early faults in complex industrial processes.

Early fault detection has significant practical application value in many industrial fields. In chemical production processes, any failure at a given stage can lead to severe safety incidents and economic losses. The proposed model can be used to detect early faults in critical equipment, such as reactors and separation devices, preventing downtime and accidents caused by equipment failure. For example, by monitoring the temperature and pressure data of a reactor, the proposed model can identify anomalies at an early stage of failure, allowing timely preventive measures to be taken to ensure production safety.

In automated manufacturing processes, equipment failure can lead to production line stoppages, affecting production efficiency. The proposed model can be used to detect early faults in various mechanical equipment on the production line, enhancing equipment maintenance management. For example, in automotive manufacturing, by monitoring the motion trajectories and current data of robotic arms, the proposed model can provide early warnings of mechanical failures, reducing downtime and maintenance costs.

## 6. Conclusions

Traditional spatio-temporal analysis methods have the problem of poorly correlating high-frequency temporal dynamics with space. Therefore, a WT-based STHM is proposed in this paper to improve the accuracy and sensitivity of early fault detection in complex industrial systems. The data were denoised through the WT, and the PCA method was used to construct the principal subspace. Moreover, the CUSUM and MD were used to build the hybrid model, which greatly enhanced fault detection performance. According to the simulation of the TE process, STHM outperforms the PCA and STN methods in both the FDR and FAR, indicating the great potential of STHM in actual industrial applications. However, the high sensitivity of STHM may lead to false alarms. Future research should focus on the optimization of model performance and improvement of algorithm robustness so that more comprehensive technical innovations and applications can be achieved.

## Figures and Tables

**Table 1:** Main fault types of the TE process.

Fault Number	Variable Name	Type
1	Abnormal feed ratio	Step change
3	Abnormal feed temperature	Step change
4	Abnormal temperature of reactor cooling water	Step change
8	Abnormal feed composition	Random variable
12	Abnormal inlet temperature of condenser cooling water	Random variable
13	Abnormal reaction kinetics constants	Slow drift
14	Abnormal reactor cooling water valve	Sticking
15	Abnormal condenser cooling water valve	Sticking

**Table 2:** Fault detection rates and product false alarm rates for different detection methods.

Fault Number	FDR(%)				FAR(%)			
	PCA-T^2^	PCA-SPE	STN	STHM	PCA-T^2^	PCA-SPE	STN	STHM
1	99.5	99.8	99.6	99.8	0	10.6	2.5	0
3	2.3	19.1	36.2	80.6	0	23.1	4.4	6.9
4	41.9	100.0	98.6	99.9	0.6	16.9	2.5	0
10	41.6	71.3	75.6	99.4	0	11.9	10	0
12	98.9	98.6	98.4	99.4	1.3	18.1	10.6	3.1
13	95.1	95.8	94.2	92.6	0	7.5	9.4	3.1
14	99.9	99.9	98.2	98.6	0.6	19.4	4.8	1.3
15	5.0	20.9	38.0	84.6	0	11.9	46.9	6.3

## Data Availability

Data are contained within the article.
